# Tumor ablation: emerging uses, challenges, and strategic implementation. A green paper by the Network of Expertise in Cancer (JANE-2), High Tech Medical Resources, network on Physical Methods of Tumor Ablation

**DOI:** 10.2478/raon-2026-0030

**Published:** 2026-06-26

**Authors:** Julie Gehl, Philippe L Pereira, Colin P Cantwell, Frédéric Deschamps, Anja Kocijancic, Nina Schmidt, Rok Dezman, Greta Chlebopasevienė, Andrei Roman, Maja Cemazar, Gregor Sersa

**Affiliations:** 1 Department of Clinical Oncology and Palliative Care, Zealand University Hospital, Roskilde, Denmark; 2 Department of Clinical Medicine, Faculty of Health and Medical Sciences, University of Copenhagen, Copenhagen, Denmark; 3 Center of Radiology, Minimally Invasive Therapies and Nuclear Medicine, SLK-Kliniken GmbH, Heilbronn, University of Heidelberg and University of Tübingen, Heilbronn, Germany; 4 Department of Radiology and University College Dublin, St. Vincent’s University Hospital, Dublin, Ireland; 5 Gustave Roussy, Departement Anesthésie, Chirurgie et Interventionnel (DACI), Service de Radiologie Interventionnelle, Villejuif, France; 6 Institute of Oncology Ljubljana, Ljubljana, Slovenia; 7 Institute of Radiology, University Medical Centre Ljubljana, Ljubljana, Slovenia; 8 Faculty of Medicine, University of Ljubljana, Ljubljana, Slovenia; 9 Department of Oncology and Hematology, Lithuanian University of Health Sciences Hospital, Kaunas Clinics, Kaunas, Lithuania; 10 Department of Radiology, The Oncology Institute “Prof. Dr. Ion Chiricuţă” Cluj-Napoca, Cluj-Napoca, Romania; 11 Department of Radiology, “Iuliu Hațieganu” University of Medicine and Pharmacy, Cluj-Napoca, Romania

**Keywords:** cancer ablation, high-tech innovations, emerging applications, regulatory issues, ethical issues

## Abstract

The field of oncology has witnessed remarkable progress with the integration of high-tech innovations in tumor ablation. Tumor ablation therapies, such as radiofrequency ablation (RFA), microwave ablation (MWA), cryoablation (Cryo), irreversible electroporation (IRE), and electrochemotherapy (ECT) have evolved beyond conventional boundaries, offering patients less invasive, highly targeted therapeutic options. Since it works across cancer histologies, tumor ablation is being integrated into cancer care at several levels. Tumor ablation is even considered curative in selected patients with small primary or secondary tumors, e.g. in the liver. Furthermore, it is central in treatment of oligometastatic disease or oligoprogression. Finally, ablation is widely used for symptomatic relief when tumors lead to symptoms affecting quality of life. Knowledge about ablative therapies and their inclusion at multidisciplinary team decision making enables effective and more personalized treatment for patients. This review synthesizes emerging applications of these therapies, focusing on artificial intelligence-driven personalization, robotic-assisted precision, and hybrid models combining ablation with systemic treatments, immunotherapy or targeted drug delivery. It also discusses the infrastructural, educational, regulatory, and ethical challenges that influence the clinical adoption of such treatments. Finally, the review presents strategic recommendations for integrating advanced ablation therapies into healthcare systems while ensuring equity, patient trust, and global accessibility.

## Introduction

### Ablation therapies

Cancer treatment as a global health problem is driven by technological advances in drug discovery and in novel medical devices.

While the biological basis and characteristics of cancer are better understood today and are driving medical oncology towards targeted therapies and personalized medicine, ablative therapies, which can be used instead of surgery, are increasingly in demand. Their development has accelerated significantly in recent decades^1^, with the aim to be tumor-specific, while sparing healthy tissue. With several developments ablative therapies have become more effective, patient-oriented and less invasive treatments which in turn are increasing their clinical relevance. Furthermore, developments that integrate artificial intelligence^2^, robotics^3^, and realtime imaging^4^ have expanded the scope and effectiveness of ablation therapies, making ablation an essential part of modern oncology care.

**FIGURE 1.**
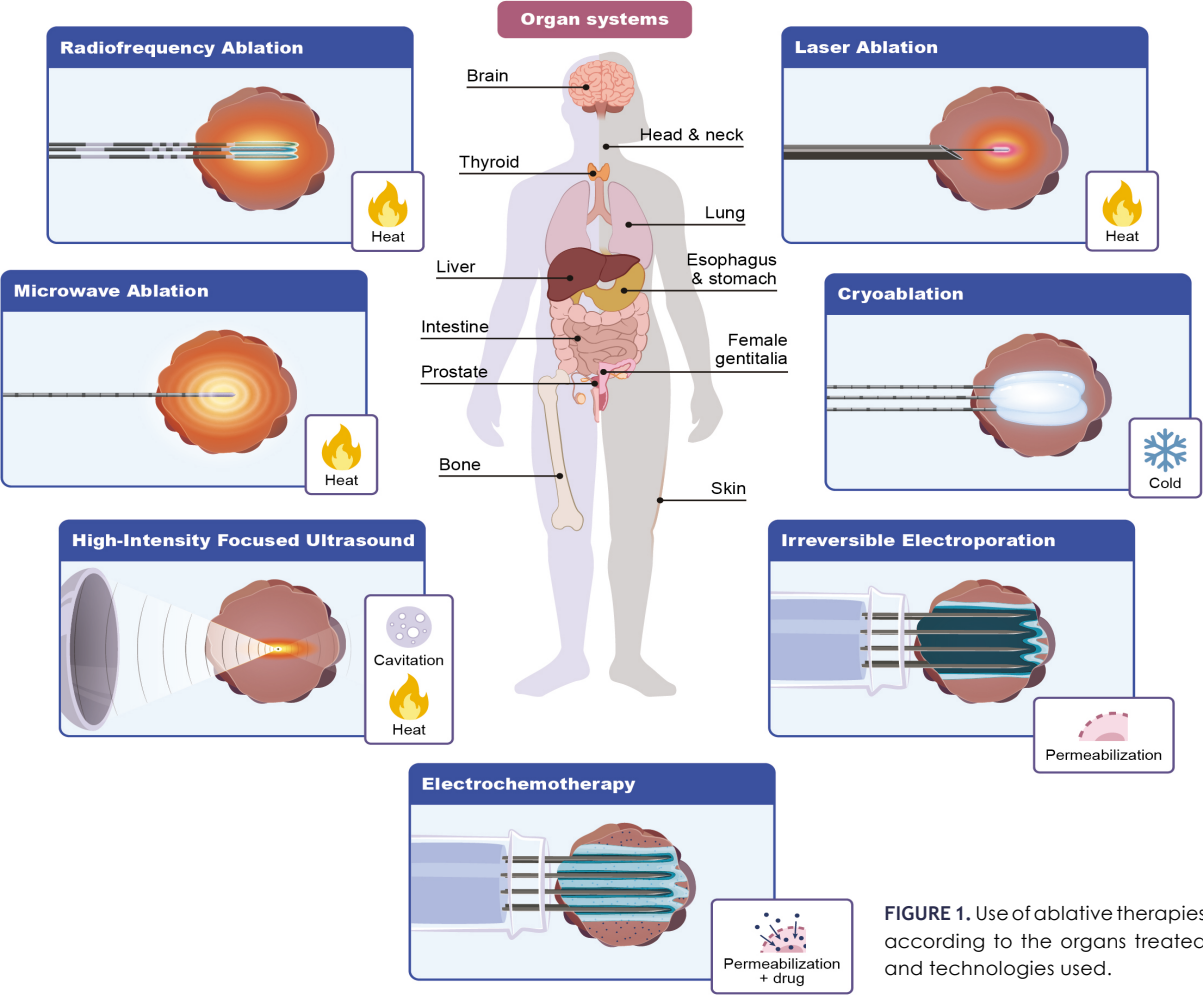
Use of ablative therapies according to the organs treated and technologies used.

The best-known ablation modalities include radiofrequency ablation (RFA), microwave ablation (MWA), cryoablation (Cryo), high-intensity focused ultrasound (HIFU), laser ablation, irreversible electroporation (IRE) and electrochemotherapy (ECT), each of which has different mechanisms of action and applications. An overview of the most used ablative therapies can be found in Table 1, Figure 1.

**TABLE 1.** Common ablative therapies in oncology with a brief description of their mode of action, common applications, advantages and disadvantages. Most of them are considered thermal ablations, except for irreversible electroporation and electrochemotherapy

Ablative therapy	Mode of action	Common applications	Advantages	Disadvantages
**Radiofrequency ablation (RFA)**	High-frequency alternating current generates heat, causing irreversible coagulative necrosis through protein denaturation	Liver, kidney, lung and bone tumors	Minimally invasive, widely available, effective for small tumors	Limited effectiveness near large blood vessels (heat sink effect), not ideal for cystic tumors. Extent of ablation area not visible in real time
**Microwave ablation (MWA)**	Electromagnetic waves agitate water molecules, generating heat and inducing irreversible coagulative necrosis	Liver, kidney lung and bone tumors	Faster and larger ablation zones than RFA, less affected by heat sink	Risk of overheating nearby critical tissues. Extent of ablation area not visible in real time
**Cryoablation (Cryo)**	Freezing and thawing causes ice crystal formation and cell rupture leading to coagulative necrosis	Prostate, kidney, bone, desmoid and lung tumors	Good real time visualization of ablation area with imaging, less pain post-procedure	Longer procedure time, risk of cryo-shock in liver ablation, costs
**Laser ablation**	Concentrated light energy heats and destroys target tissue by coagulative necrosis	Brain tumors, liver metastases, dermatology	Precise and controllable,	Very limited penetration
**Irreversible electroporation (IRE)**	Electric pulses create permanent nanopores in cell membranes, inducing apoptosis	Tumors near critical structures (e.g., bile ducts in liver, pancreas, prostate)	Non-thermal, spares connective tissue and vessels	Longer procedure time and general anesthesia recommended
**Electrochemotherapy (ECT)**	Electric pulses increase cell membrane permeability, enhancing drug uptake, leading to different types of cell death depending on the concentration of the drug	Cutaneous, subcutaneous, mucosal and deep-seated tumors, potentially pancreatic cancer	Highly effective for superficial tumors, low systemic toxicity, spares connective tissue and vessels	Bimodal treatment, drug and application of pulsed electric field

This report summarizes the latest research findings and developments in the field of cancer ablation, analyzes the emerging clinical applications and provides insights into the strategic integration of these technologies through EU Joint Action Networks of Expertise on Cancer (JANE-2) project into various areas of cancer treatment.

### The JANE-2 project

JANE-2 project aims to build networks of expertise in cancer care (more on https://jane-project.eu/)^5^, that will share best practices and knowledge, and ensure accessible, cutting-edge treatment for every patient, regardless of their location. The project is organized into work packages, and work package 10 is dedicated to hi-tech medical resources. This package is divided into 7 domains, each focusing on its own technology – from innovative surgery to ex-vivo testing of agents.

**FIGURE 2.**
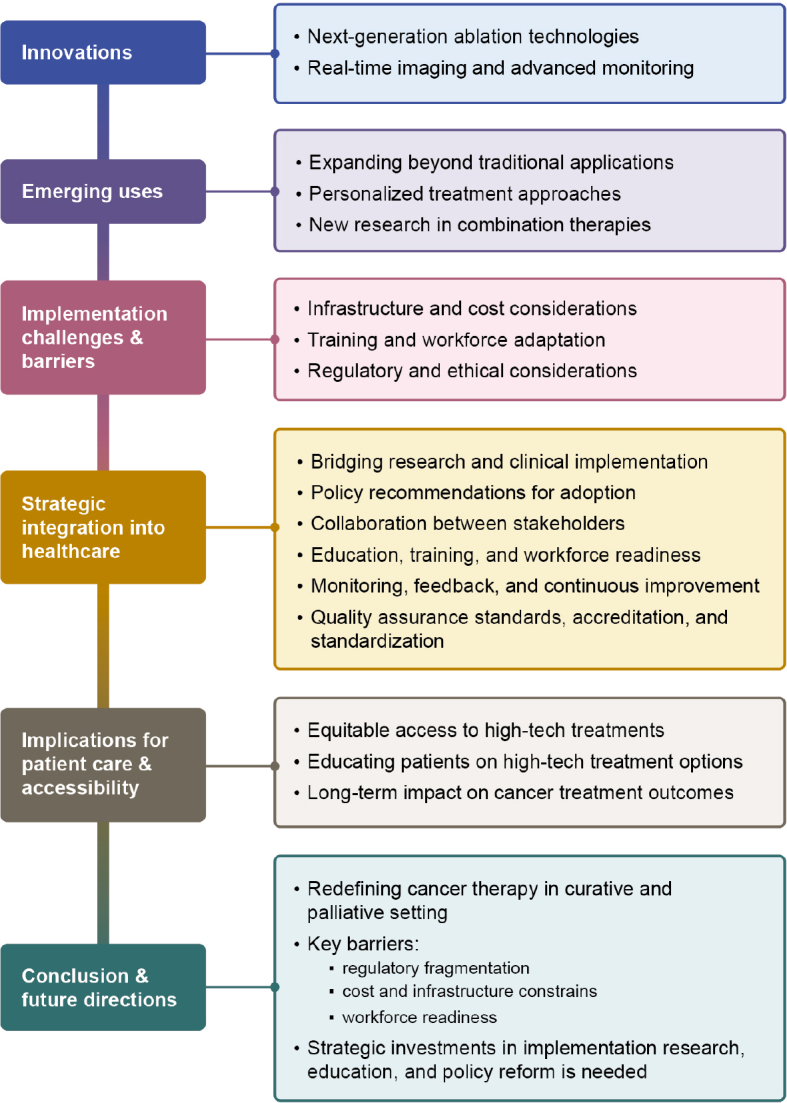
Schematic and graphical presentation of the aims of the Physical Methods of Ablation Domain of the JANE-2 project, which are discussed in this review for broader application of ablative therapies in treatment of cancer throughout Europe.

One of these domains is focusing on physical ablation therapies and is aiming to make a significant impact on clinical practice guidelines for ablation therapy across Europe. It will cover all thermal and non-thermal ablative therapies currently used and applied for cancer treatment. Our main goals and activities include supporting centers in integrating innovative therapies, enhancing medical education, raising patient and public awareness, and ensuring the network’s visibility and sustainability through dissemination and communication activities. Therefore, this narrative review reflects the view of the members of this domain and indicates specific points that need to be addressed. The points and topics of discussion in this review are summarized in this graphical presentation in Figure 2.

### Clinical need

Oligometastatic disease and oligoprogression or oligopersistence are laid out in a recent article by Guckenberger *et al*.^6^ Here, a novel paradigm is supported, where patients are considered salvageable in case metastases are limited (oligometastatic disease), or there is progression only in one or few lesions during or following systemic treatment (oligoprogression), or one or few metastatic lesions persisting after otherwise successful systemic treatment.

These lesions may be treated by a variety of techniques, including surgery, radiotherapy or physical methods of ablation. Clinical examples include stereotactic radiotherapy of brain metastases^7^, surgical removal of lung metastases.^8^ Physical ablation of liver metastases is widely and frequently used^9^, as is ECT for cutaneous metastases.^10^ Physical ablation may also be relevant for patients with symptomatic metastases, where treatment is palliative.

Similarly, the role of physical ablation is well established as a curative treatment for small primary tumors such as hepatocellular carcinoma^11^, renal cell carcinoma^12^, and lung cancer.^13^ It is generally employed for patients who are poor surgical candidates due to comorbidities and inaccessible tumors, as well as for patients who refuse surgery. For some tumors such as hepatocellular carcinoma, physical ablation is considered as a first treatment option.^11^

### Multi-disciplinary team decisions

The multidisciplinary teams (MDTs) are now a fixture in modern cancer care. Here, treatment decisions may be made with expertise across specialties, and combining systemic and local treatment strategies.^14^ In an expanding treatment landscape, the MDTs become increasingly important in keeping overview of all treatment possibilities for patients and offering personalized treatment solutions. Knowledge of ablation methods, including different therapies such as stereotactic body radiotherapy that has very similar indications and specific advantages of these, as well as treatment results for different clinical presentations should be available at the MDT. This may require expansion of the team to include further specialists including interventional radiologists and an overview of referral possibilities.

## Emerging high-tech innovations in ablation therapy

### Technological advancements of local ablative therapies

Recent developments in applicator design, energy control, *in vivo* treatment prediction, guidance, and monitoring have dramatically improved the efficacy, predictability and adaptability of traditional ablation therapies such as RFA and MWA. Modern systems enable fine-tuned control of energy parameters (e.g. amplitude, duration, frequency), allowing physicians to precisely adjust the size and shape of the ablation zone to the dimensions of the tumor.^15–19^

The integration of artificial intelligence (AI) into these platforms enables real-time adjustment during the procedure and improves the precision with which the energy is delivered to the target tissue.^2^ Many instruments now integrate near real-time monitoring and adaptive software that adjusts energy delivery based on feedback on tissue response (temperature, electrical conductivity, etc.).^4^

Machine learning models trained on large datasets of clinical imaging and procedural outcomes are increasingly being used to simulate ablation zones, optimize ablation duration and intensity, and predict tissue response based on perfusion or cell density. This data-driven personalization increases both the safety and efficacy of ablation, especially for tumors with heterogeneous composition or those located in anatomically complex regions.

Robotic assistance may further increase the accuracy of the procedure and the consistency of the operator. Robotic platforms have proven successful in the guidance of placement of RF-applicators or MW-antennas for percutaneous ablation of tumors in the liver, an area traditionally considered difficult to target due to respiratory motion or proximity to large vessels and critical organs. Robotics reduce the duration of the procedure, operator fatigue and inter-operator variability while ensuring high reproducibility.^20^

In addition, the future of ablation lies in multifunctional approaches that combine local energy delivery with local or systemic delivery of therapeutic agents.

### Real-time imaging and advanced monitoring

The success of any ablative therapy depends on accurate visualization of tumor margins and monitoring of treatment effects. To this end, advances in real-time imaging and intraoperative monitoring play a crucial role in improving the precision and safety of the therapy.

Computed tomography is currently the most widely used modality for intraprocedural confirmation of the ablation zone. Contrast-enhanced CT (CECT) with image fusion, ideally supported by ablation confirmation software, is increasingly emerging as a standard approach for ablation zone confirmation.^21^ Magnetic resonance imaging (MRI) is now routinely used for high-resolution anatomical and functional imaging, particularly in the brain^22^, liver^23^ and prostate.^24^ MRI-guided ablation allows not only accurate targeting but also real-time thermometry^19^,^25^, which provides immediate feedback on tissue temperature and the extent of coagulation when using tumor ablation therapies that relies on tumor destruction by coagulative necrosis. This is particularly important in organs where there is a risk of thermal damage and where preservation of function is critical.

Other imaging techniques, such as contrast-enhanced ultrasound (CEUS), have become an important tool for assessing the effectiveness of treatment in the field. CEUS is used in liver and kidney tumors to assess residual perfusion immediately after ablation, thus facilitating the decision for further treatment.^26^ In addition, PET-CT imaging may improve tumor localization^27^, particularly in metabolically active cancers, and is currently being explored to monitor early metabolic changes after ablation that indicate treatment response or failure.^28^

Technological innovations in ablation therapy are rapidly changing the therapeutic landscape in interventional oncology. The convergence of artificial intelligence (AI), isotropic CT and MR imaging, real-time image fusion, robotics, intelligent energy delivery and near real-time monitoring is enabling highly personalized, precise, safe and adaptive interventions that go far beyond the traditional applications of local ablation therapies. These new tools not only improve the clinical and patients reported outcomes and safety of the procedure, but also open new avenues for integrating ablation with systemic and other targeted therapies. With advances in research and clinical validation, high-tech local tumor ablation therapies will become a central pillar of multidisciplinary cancer treatment.

## Emerging uses of ablation

### Expanding beyond traditional applications

Historically, ablative therapies RFA, MWA and Cryo were mainly used in patients who were not candidates for surgical resection due to tumor location, concomitant diseases or limited performance status.^29–31^ However, the role of these therapies is rapidly evolving. They are now being investigated and used as first-line therapies for selected early stage solid tumors, including hepatocellular carcinoma (HCC)^32^, renal cell carcinoma (RCC)^33^ and non-small cell lung cancer (NSCLC)^34^ and extraabdominal desmoid tumors.^35^ For example, percutaneous RFA and MWA have shown non-inferior local control rates compared to surgical resection in small-size colorectal liver metastases^36^, small HCC (≤ 3 cm) and T1a renal cancer with significantly reduced perioperative mortality and morbidity.^37,38^

**FIGURE 3.**
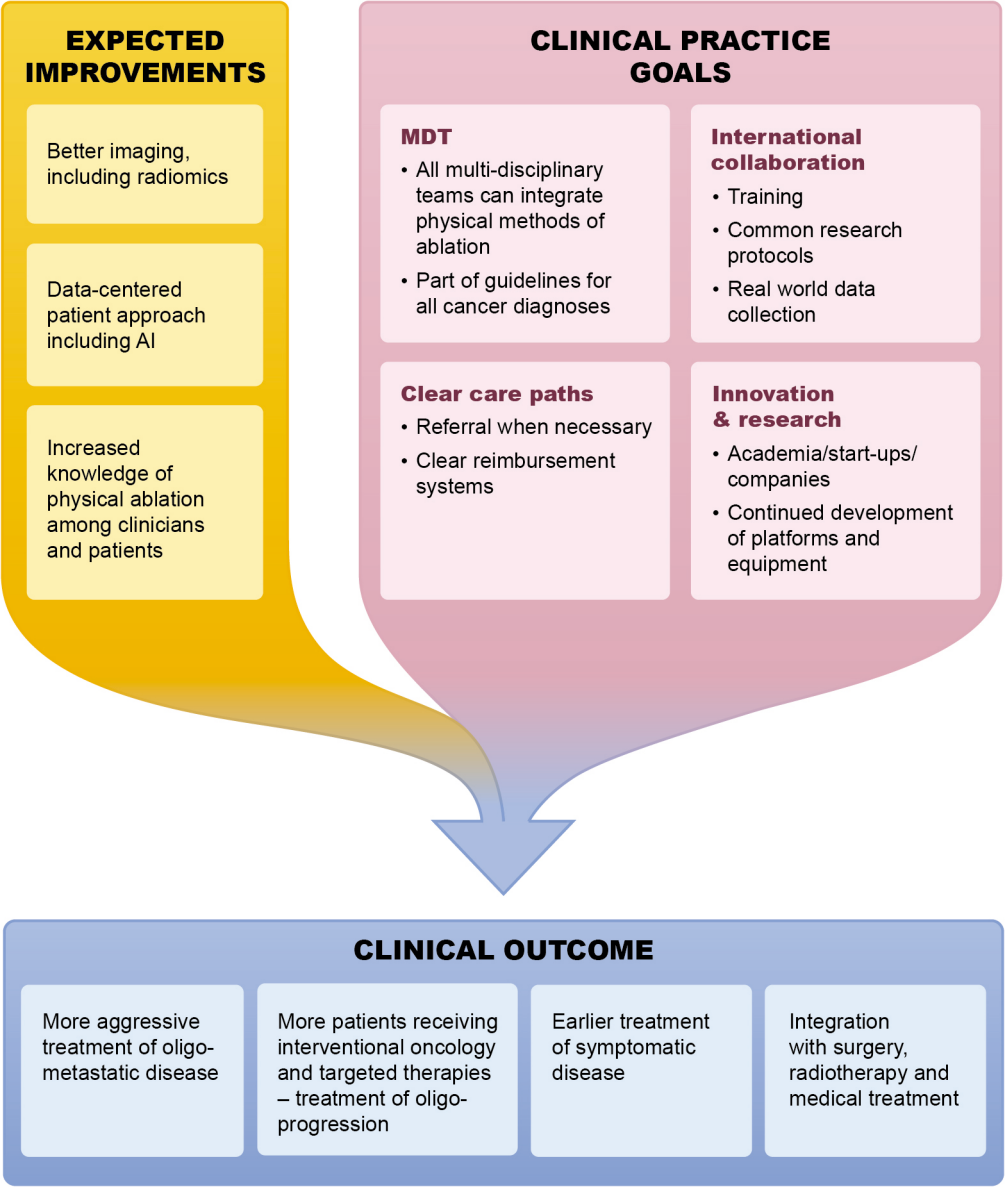
Envisioned development and integration of physical methods of ablation into cancer care paths.

This shift is supported by high-resolution imaging guidance, real-time monitoring capabilities (e.g. with contrast-enhanced ultrasound, CT/MRI fusion and near real time MR imaging) and thermal dose modeling to improve precision. Not only for elderly or frail patients, but for selected patients of all ages, these less invasive procedures offer shorter hospital stays, faster recovery times, better quality of life and a lower risk of surgical complications^39^, features that are crucial for the management of age- and comorbidity-related oncology patient groups (Figure 3). If the patients can be treated with minimally invasive local ablative therapies, there is also societal benefit due to decreased health care cost, use of hospital facilities, as well as health care staff time.

High-tech ablation is also used to target recurrent or oligometastatic lesions, particularly of lung^40^, kidney^41^ and metastatic colon cancer.^42^ Ongoing studies indicate that local ablation of oligometastases can improve progression-free and overall survival in selected patients.^32^,^33^

Whereas as ECT primarily uses the chemotherapeutic drug bleomycin, recent advances show that using calcium leads to similar results as well as immunological responses.^43–45^

### Personalized treatment approaches

The integration of precision medicine principles with ablation is accelerating rapidly. Advances in multi-omics technologies (genomics, transcriptomics, proteomics) combined with AI and machine learning are being used to optimize patient selection and treatment planning.^46^ AI-driven radiomics can analyze imaging data to predict tumor response, identify anatomical risk zones and optimize applicator positioning for maximal tumor coverage while sparing adjacent critical structures.^47^ In addition, biological markers and/or algorithms can stratify patients based on tumor biology and immune microenvironment and identify those who are likely to respond to thermal or non-thermal ablation therapies.^48–51^

Furthermore, predictive models can estimate the probability of local recurrence or the need for adjuvant therapy after ablation.^52–54^ Clinical decision support systems (CDSS) can be integrated into the treatment suites and enable real-time synthesis of imaging, histopathology and patient-specific factors to dynamically adapt treatment strategies and improve personalization and safety.^55^

### New research in combination therapies

An area of growing interest is the synergistic action of ablation and immunotherapy.^56^ Ablative therapies, either thermal or non-thermal, particularly those that can induce immunogenic cell death (ICD), can transform tumors into *in situ* vaccines.^57^

For example, thermal ablation causing coagulative necrosis can release damage-associated molecular patterns (DAMPs), such as heat shock proteins and tumor-associated antigens (TAAs) and stimulate dendritic cell activation and promote T cell infiltration.^58^ Similarly, IRE and ECT, which preserve the extracellular matrix and vasculature, have the same *in situ* vaccination effect and support a tumor-specific immune response without excessive inflammation.^59–61^

This has led to several ongoing clinical trials investigating the use of ablation in combination with immune checkpoint inhibitors (ICIs) such as anti-PD-1 or anti-CTLA-4.^62^ Data from early phase clinical studies^43,44^ indicate an enhanced systemic anti-tumor immune response, particularly in immunologically “cold” tumors such as pancreatic or colorectal cancer^63^ and in some cases also melanoma.^64,65^

Furthermore, ablation probes can be functionalized to simultaneously deliver chemotherapeutic agents^43^, cytokines^66^ or nanoparticles^67^ during energy delivery. For example, liposomal doxorubicin or IL-12-encoding plasmids can be released locally in response to temperature or the electric field, concentrating the drug’s effect on the tumor and reducing systemic toxicity.^68,69^

These innovations emphasize the paradigm shift from ablation as merely a cytoreductive tool to a multifunctional platform that has the potential to reshape the oncologic treatment landscape through precision targeting, immunoactivation, and therapeutic molecule delivery.

## Implementation challenges and barriers

### Infrastructure and cost considerations

Despite the transformative potential of high-tech ablation therapies, access remains uneven due to significant infrastructural and economic barriers within Europe. Many advanced ablation systems, particularly those with artificial intelligence, robotic guidance or real-time imaging come with high upfront costs. Moreover, in some countries, physical ablation techniques are not recognized as standard treatment, leading to a lack of state reimbursement for the necessary devices and consumables, which must instead be covered by hospital resources. These financial demands are particularly burdensome for facilities in resourcepoor settings, where healthcare budgets are already constrained and access to specialized medical staff may be limited.

To mitigate these inequalities, strategies such as the establishment of central competence centers and the use of mobile ablation units have been proposed. This can extend the reach of services without the need to duplicate costly infrastructure in every location. In addition, international collaborations/twinning’s and technology transfer agreements can support capacity building in underserved regions.

Economic viability remains an important consideration. Cost-effectiveness analysis comparing advanced ablation therapies with conventional surgical or radiotherapeutic approaches are limited but necessary. These analyses should consider not only direct costs, but also long-term outcomes, shorter hospital stays and improved quality of life. Therefore, the patient’s reported outcomes should be essential in foreseen clinical trials. In addition, reimbursement policies and insurance frameworks often lag behind technological advances, further impeding widespread adoption. Collaborative health technology assessment, public awareness and proactive political engagement are essential to align innovative ablative therapies with health system priorities.

Another barrier for uniform use of ablative therapies across Europe is different reimbursement systems between different EU countries, including sometimes complicated procedures for reimbursement. The cost of the probes for different ablative therapies varies, but broader use of these techniques should level out the costs between different local ablative therapy approaches. Furthermore, the EU must approach this problem with strategy implementation of unified code for reimbursement in EU countries. This would increase the accessibility of the tumor ablative therapies to broader patient population and level out the disparities in reimbursement between the countries.

### Training and workforce adaptation

The successful use of high-tech ablation requires staff who are qualified in both clinical and technical areas. Physicians must be proficient in interpreting advanced imaging, operating equipment and, increasingly, integrating AI tools into decision-making processes. This often requires multidisciplinary procedural training.

To implement this, curricula are evolving to include simulation-based platforms, virtual reality modules and distance learning opportunities. These tools facilitate safe, repeatable practice and allow for broader dissemination of expertise beyond academic centers. Training in multidisciplinary teams involving interventional radiologists, oncologists, surgeons, physicists and biomedical engineers, reflect the collaborative nature of modern ablation therapies. The guidelines and Standard Operating Procedures should be set on an international and interdisciplinary level. Ideally, these learning courses should be certified by Continuing Medical Education (CME) and EU micro-credential for lifelong learning. Valuable experience in education is gained in training by professional societies like Cardiovascular and Interventional Radiological Society of Europe (CIRSE)^70^ and European Society of Surgical Oncology (ESSO)^71^ that are following curricula and have an examination or a certification endorsed by European Union of Medical Specialist^72^ and European Society of Radiology.^73^ Training in capabilities of ablative methods must be included in training and continued education of oncologists.

However, the introduction is not only a technical challenge but also requires different thinking in care paths. Institutional leadership, supportive regulators and a strong emphasis on continuing professional development and mentoring are needed to overcome these barriers.

### Regulatory and ethical considerations

The integration of AI, robotics and real-time data analysis into ablation therapy involves complex regulatory and ethical aspects. Unlike conventional tools, intelligent systems evolve based on data inputs, raising concerns about the transparency, bias and accountability of algorithms. It is crucial for patient safety and public trust that these tools are rigorously validated, explainable and subject to external oversight.

Regulators need to adapt quickly to the changing landscape and develop frameworks that balance innovation and safety. Harmonization of standards across countries is necessary to prevent fragmented implementation and facilitate crossborder cooperation. Manufacturers must also be encouraged to use interoperable data formats and transparent reporting practices to support verifiability and post-market surveillance.

## Strategic integration of high-tech ablation into healthcare

### Bridging research and clinical implementation

To ensure rapid and efficient translation of research findings into clinical practice, oncology centers must cultivate a robust innovation ecosystem that bridges the gap between the bench and the bedside. This requires structured translational pipelines that integrate preclinical validation, first-in-human studies and adaptive implementation models in routine oncology care.

Key enablers include partnerships between academia and industry, dedicated translational research teams and innovation incubators in hospitals. These platforms help researchers overcome regulatory, financial and logistical hurdles to bring promising technologies such as AI-guided ablation, robotic navigation systems and smart energy delivery devices to patients faster.

Early technology adopters often referred to as centers of excellence serve as test institutes for iterative technology refinement and validation in the field. They also act as centers for training clinicians, gathering evidence from the field, and conducting translational research, which is essential for scaling successful interventions in various healthcare settings.

In addition, adaptive clinical trial designs, such as umbrella clinical trials and embedded implementation studies enable real-time evaluation of safety, efficacy and workflow integration. Embedding such innovations in learning healthcare systems enables continuous feedback loops between research, care and policy adaptation.

### Collaboration between stakeholders

The strategic integration of high-tech ablation into healthcare systems depends on the collaboration of multiple stakeholders. Each stakeholder group, researchers, clinicians, regulators, payers, technology developers and patients, offers unique perspectives that must be harmonized for effective and ethical implementation.

Interdisciplinary advisory tumor boards should be set up to assess not only the clinical and economic value of new ablation technologies, but also their social impact. These boards can advise on clinical indications, cost-effectiveness thresholds, training standards and monitoring frameworks. An accreditation system in interventional radiology has been established.^74^

Public-private partnerships (PPPs) can accelerate the scaling of innovation by aligning academic ingenuity with industrial production and commercialization capabilities. Successful PPPs often include risk-sharing funding models, joint technology development and shared intellectual property management frameworks that incentivize collaboration.

Professional societies play a crucial role in dissemination and standardization. They can develop clinical practice guidelines, training programs, certification programs and multicenter registries that support the evidence-based use of advanced ablation tools.^75^

Patient and their care-givers involvement is crucial from early conception to late implementation. Co-developing tools with patients ensures usability and uptake, while patient-reported outcomes and patient decision aids help to align implementation with patient values, improve satisfaction and reduce disparities.

### Education, training, and workforce readiness

Efforts by national radiological societies are needed to assess access to physical ablation techniques, as in some countries their availability is limited to only a few hospitals. In such settings, training opportunities are also scarce, therefore, collaboration between national and European societies, such as CIRSE, could help establish a framework for targeted international educational programs.

A successful transition to high-tech ablation requires significant investment in workforce development. As technologies become more complex, there is a risk that the gap between technical skills and clinical knowledge will widen. It is crucial to train a new generation of clinicians who are not only proficient in traditional oncology, but also fluent in digital tools, robotics and bioengineering principles.

Collaboration with academic centers and engineering faculties can promote dual degree programs, innovation fellowships and interdisciplinary internships that prepare clinicians to lead the digital transformation in oncology.

In addition, support staff including nurses, technicians and data analysts must be able to operate and interpret advanced systems. The formation of multidisciplinary implementation teams promotes collaborative care models and ensures that innovations are smoothly embedded into clinical workflows.

### Monitoring, feedback, and continuous improvement

Post-implementation monitoring is critical to ensure that new technologies deliver what they promise. Robust data infrastructures should be put in place to track results in practice, identify adverse events and assess long-term cost-effectiveness.

Healthcare institutions should introduce dashboards with performance indicators tailored to specific ablation therapies. These may include metrics such as procedure duration, tumor removal accuracy, patient recovery times and reintervention rates.

Health Technology Assessment (HTA) bodies need to introduce dynamic review cycles that take into account real-world evidence, technological iterations and patient feedback. Adaptive reimbursement models, such as value-based pricing and outcomes-based contracts, can be linked to ongoing performance data.

Finally, international cooperation is essential for harmonizing standards, sharing best practices and building global learning networks. Initiatives such as joint registries, benchmarking consortia and cross-border pilot projects can accelerate global access to safe and effective high-tech ablation.

### Quality assurance standards, accreditation and standardization

The increasing demand for and adoption of ablative therapies highlight the need for robust quality assurance frameworks to ensure safe and effective clinical implementation. The CIRSE Standards of Quality Assurance in Interventional Oncology^76^ provide an internationally recognized framework defining the organizational infrastructure, multidisciplinary integration, procedural expertise, and clinical governance required for high-quality interventional oncology services. The Standards are supported by over 40 national and international societies, including the European Cancer Organization (ECO).^77^

These standards outline key requirements such as participation in multidisciplinary tumor boards, appropriate patient selection processes, minimum procedural experience, and the availability of specialized staff and facilities. By establishing clear benchmarks for service organization and clinical practice, the standards aim to promote consistency, safety, and accountability across institutions providing ablative therapies.

Building on this framework, the International Accreditation System for Interventional Oncology Services (IASIOS)^74^ was developed to operationalize these standards through an international accreditation and peer-review process. IASIOS provides an external evaluation of institutional practice based on predefined quality indicators derived from the CIRSE standards.

Accredited centers undergo structured assessment of their clinical pathways, infrastructure, multidisciplinary collaboration, procedural volumes, and quality management systems.

In addition, the standardized treatment approaches for effective and safe execution of the procedures are available for different ablative techniques. For example, the emerging ablative technique such as electrochemotherapy, the preparation of standard operating procedures^78^ has vastly contributed to its adoption and spread through the European cancer centers. Furthermore, the clinical data repositories from multiple centers have proved useful for increased evidence of the treatment safety and effectiveness. Again, the InspECT database^79^ has proved of great importance and helpful to provide the multicentric clinical results.

## Implications for patient care and accessibility

### Equitable access to high-tech treatments

While high-tech ablation therapies are clinically promising, they also risk deepening existing disparities in cancer care if accessibility is not proactively addressed. Geographic, socioeconomic and infrastructure inequalities may prevent timely access to cutting-edge therapies, particularly in rural, low-income or resource-limited regions.

To improve affordability, tiered pricing structures, insurance reimbursement schemes and public subsidies are crucial. Partnerships with global health organizations such as the WHO and the International Atomic Energy Agency (IAEA), CIRSE and other scientific organizations can support knowledge transfer, capacity building and infrastructure provision in low- and middle-income countries.

### Educating patients on high-tech therapy options

The complexity of high-tech ablation therapies requires sound patient education strategies that ensure informed decision-making and autonomy. Patients need clear, non-technical explanations about the mechanism of action, expected outcomes and potential risks of ablation therapies.

Frameworks for shared decision-making are being established, supported by decision aids, visual aids and multilingual resources that take account of different levels of health literacy. Involving caregivers and patient advocates in consultations further strengthens trust and treatment adherence.

The digital transformation in healthcare also requires patients to have the necessary digital skills to deal with remote monitoring systems, virtual consultations and AI-supported risk assessments. Digital literacy programs led by community health workers or integrated into survivorship clinics can help bridge this gap.

### Long-term impact on cancer treatment outcomes

Initial clinical data suggest that high-tech ablation therapies are safe and may contribute to improved local tumor control with lower recurrence rates and thus with shorter hospital stays^80^, especially when used early or in combination with systemic therapies. In addition, outpatient models enabled by miniaturized portable devices would reduce the overall burden of treatment, reduce inequity in access to the treatment, improve patient satisfaction and reduce pressure on hospital resources.

To fully understand the long-term clinical and economic impact, longitudinal data needs to be collected through large prospective registries, real-world data platforms and multi-center clinical trials. Integration with electronic health records (EHR) and digital biomarkers will support continuous learning and adaptive treatment algorithms.^81^

Future research should focus on the identification of predictive biomarkers for response to ablation, the optimization of sequential and combination therapies (e.g. with immunotherapy or targeted agents) and the refinement of personalized ablation planning using genomic, radiomic and immunoprofiling data.

## Conclusion and future directions

High-tech innovative local ablative therapies are changing the field of cancer ablation and opening new possibilities for precise, safe, patient-centered and minimally invasive interventions. By integrating AI, robotics, advanced imaging, biomarkers and bioelectronic interfaces, these therapies promise to redefine cancer therapy in curative and palliative settings. However, their widespread adoption depends on overcoming key barriers: regulatory fragmentation, cost and infrastructure constraints and workforce readiness. Strategic investment in implementation research, education and policy reform is needed to ensure fair and efficient integration of these therapies into clinical practice.

The future lies in intelligent, adaptive systems that can learn from real-time data, in miniaturized and portable ablation devices for decentralized care and in multimodal treatment platforms that combine ablation with data obtained from e.g. genomics/proteomics/radiomics, as well as systemic treatments, such as immunotherapy.

A joint approach is essential. Policymakers must create an enabling environment, clinicians and scientists must drive ethical and evidence-based innovation, industry must prioritize interoperability and affordability, and patients must be placed at the center of technological change. Only through such cross-sector collaboration can we ensure that the promise of high-tech ablation is delivered, not as an elitist offering, but as a standard of care that improves survival, preserves quality of life and reaches everyone who can benefit from it.
